# Identification of histone 3 variant 2 interacting factors

**DOI:** 10.1093/nar/gkt1355

**Published:** 2014-01-06

**Authors:** Daniel Latreille, Lisa Bluy, Monsef Benkirane, Rosemary E. Kiernan

**Affiliations:** ^1^Laboratoire de Régulation des Gènes, Institut de Génétique Humaine, CNRS UPR1142, Montpellier 34396, France and ^2^Laboratoire de Virologie Moléculaire, Institut de Génétique Humaine, CNRS UPR1142, Montpellier 34396, France

## Abstract

The epigenome is defined as a type of information that can be transmitted independently of the DNA sequence, at the chromatin level, through post-translational modifications present on histone tails. Recent advances in the identification of histone 3 variants suggest a new model of information transmission through deposition of specific histone variants. To date, several non-centromeric histone 3 variants have been identified in mammals. Despite protein sequence similarity, specific deposition complexes have been characterized for both histone 3.1 (H3.1) and histone 3.3 (H3.3), whereas no deposition complex for histone 3.2 (H3.2) has been identified to date. Here, we identified human H3.2 partners by immunopurification of nuclear H3.2 complexes followed by mass spectrometry analysis. Further biochemical analyses highlighted two major complexes associated with H3.2, one containing chromatin associated factor-1 subunits and the other consisting of a subcomplex of mini chromosome maintenance helicases, together with Asf1. The purified complexes could associate with a DNA template *in vitro*.

## INTRODUCTION

In mammalian nuclei, DNA is packaged into chromatin, the basic unit of which is the nucleosome that is composed of 147 bp of DNA wrapped around two molecules each of histone H2A, H2B, H3 and H4 (1). Events that occur in the context of chromatin, such as DNA replication, DNA repair or transcription, necessitate a constant remodelling of nucleosomes (2). Post-translational modifications (PTM) of core histones contribute to determining states of active and repressed chromatin that can be faithfully transmitted from one generation to the next (3). The discovery of variants of histone H3 that are enriched in PTMs associated with either active or repressed chromatin has led to the notion that histone variant exchange facilitates plasticity at the nucleosome level (4). In mammals, histone H3 non-centromeric variants, H3.1, H3.2 and H3.3, differ in their chromatin deposition patterns and PTMs despite having a high degree of sequence similarity (4,5). H3.1 and H3.2, which are enriched in repressive chromatin marks, are predominantly expressed during S-Phase and deposited in DNA synthesis-coupled fashion during DNA replication and repair (replication-dependent, RD). In contrast, H3.3 is enriched in active chromatin marks and is expressed throughout the cell cycle. It is deposited in chromatin independently of DNA synthesis (replication-independent, RI) during, e.g. transcription.

Biochemical characterization led to the identification of deposition complexes that are specific for H3.1 and H3.3 histone variants (6). The trimeric chromatin associated factor-1 (CAF-1) complex, consisting of p150, p60 and p48 subunits, is associated with H3.1 and mediates DNA synthesis-dependent H3.1 deposition (7). H3.3 is deposited by at least two complexes, histone cell cycle regulation defective homolog A (HIRA) and death domain-associated protein/α-thalassaemia mental retardation syndrome X-linked (DAXX/ATRX) complexes (6,8–11). The two human isoforms of *Saccharomyces cerevisiae* H3/H4 chaperone anti-silencing factor 1 (Asf1), Asf1a and Asf1b, appear to be involved in both the RD and RI pathways (6,9,12).

Lower organisms possess a single RD H3 variant, H3.2, which is deposited by CAF-1. In contrast, mammals possess two distinct RD variants, H3.1 and H3.2, which differ by a single residue within the primary amino acid sequence. However, differences in their PTM and deposition patterns after fertilization of oocytes (9,13,14) suggest that they might not support the same function on chromatin. No deposition complex has been identified for mammalian H3.2 to date. The difference in PTM between H3.1 and H3.2 has led to the suggestion that these variants may not use the same deposition complex. To obtain insights into H3.2 deposition, for which no biochemical data are available, we used tandem affinity purification coupled to mass spectrometry (MS) to identify factors associated with eH3.2 (epitope-tagged H3.2) in nuclear extract (NE). Two major complexes associated with eH3.2 were identified: a CAF-1 complex composed of the CAF-1 p150 and CAF-1 p60 subunits, and a second eH3.2 complex consisting of mini chromosome maintenance (MCM)-2, 4, 6 and 7, together with Asf1a and/or Asf1b. Interestingly, an interaction with MCM-2/4/6/7Asf1a/Asf1b was observed for all three H3 variants tested, including eH3.3, for which no RD complex has been identified to date. Finally, both CAF-1- and MCM/Asf1-eH3.2 complexes could associate with a DNA template *in vitro*.

## MATERIALS AND METHODS

### Cell culture, transfection, transduction and sorting

The 293T cells and HeLa S3 cell were grown in Dulbecco's modified Eagle's media (Lonza) supplemented with 10% fetal bovine serum, 1% ultraglutamine (Lonza) and 1% pen/strep (Lonza) at 37°C with 5% CO_2_. For large-scale purification, S3 cells were grown in Joklik media at 37°C in the absence of C0_2_. Stable cell lines (eH3.1, eH3.2, eH3.3) were generated as described previously (15).

### Cell fractionation

Cell were lysed on ice for 7 min in cooled buffer 1 (20 mM Tris pH 7.5, 30 mM KCl, 7.5 mM NaCl, 0.5 mM dithiothreitol (DTT), 0.1 mM phenylmethylsulfonyl fluoride (PMSF) and protease inhibitor) with a final concentration of 0.1% NP-40 and 0.3 M sucrose and loaded on top of a cooled nuclei cushion (buffer 1, at 1.2 M sucrose final) before being spun at 8.5 K rpm for 20 min at 4°C in a JA.20 rotor. The cytoplasmic fraction was harvested from the top of the cushion. Purified nuclei were permeabilized in buffer 2 (1× phosphate-buffered saline, 2 mM MgCl2, 0.1 M DTT + 0.1 mM PMSF and protease inhibitor) containing 0.2–0.8% Triton X-100 for 20 min at room temperature (RT) before loading on a cooled 400 µl chromatin cushion (buffer 2 supplemented with 30% sucrose w/v) and spun at 10 000*g* for 15 min at 4°C. The nuclear fraction (upper phase) and purified chromatin (pellet) were resuspended in Laemmli buffer for analysis by immunoblotting.

### Cell synchronization

The S3 cells were grown to 30–40% confluency. A double thymidine block (2 mM final) was performed as described previously (16). After release from the second thymidine block, 10^6^ cells were fixed and permeabilized in 40% cold ethanol overnight. DNA content was analysed by measuring the incorporation of Fxcycle reagent (Invitrogen). Cells were analyzed on MACSQuant Analyzer (Milteny Biotec). Data analyses were performed on FlowJo (TreeStar Inc.).

### eH3.2-associated complex purification

HeLa NEs were prepared as described previously (17). Sequential Flag and haemagglutinin (HA) immunoprecipitations were performed on equal amounts of proteins. Silver staining was performed according to the manufacturer’s instruction (Silverquest, Invitrogen).

### Immunoprecipitation and Western-Blot

Anti-CAF p150 (Bethyl) and anti-MCM2 (Abcam) were used for IPs. The following antibodies were used for immunoblotting: anti-CAF p150 (H300), anti-CAF p60 (K-15), anti-CAF p48 (C-16), anti-MCM2 (N19) and anti-lamin A/C (F-2) from Santa Cruz Biotechnology; anti-MCM3, anti-Asf1a (C6E10) and Asf1b (C70E2) from cell signalling; MCM4, MCM5, MCM6, MCM7 from Bethyl; anti-H3 (ab1791), anti-H4 (ab31830) and anti-H2B (ab1790) from Abcam; anti-HA (HA.11 Covance); and anti-Flag (M2, Sigma). Peptides against CAF-1 (Bethyl), Flag (Sigma) and HA (Roche) were used for elution.

### Glycerol gradient

One millilitre layers of glycerol (35–15%) were loaded in Ultraclear tubes (Beckman). Ultracentrifugation was performed in a Sw55ti rotor at 40 000 rpm for 8 h at 4°C. In all, 200 µl fractions were collected from the top of the gradient. An equal volume of each fraction was analysed by immunoblotting using the indicated antibodies.

### *‘In vitro’* DNA association assay

Briefly, pUC19 was linearized using EcoRI and XmaI restriction enzymes. Phenol/chloroform-extracted DNA was refilled with biotin-14-dATP using Klenow enzyme (5′-, 3′- exonuclease activity). DNA was coupled to beads (Dynabeads) in binding buffer (10 mM Tris pH 7.5, 2 M NaCl, 1 mM EDTA). Assays were performed using the same amount of DNA in a final reaction volume of 60 µl (40 mM Hepes pH 7.6, 5 mM MgCl2, 2.5 mM DTT, 20 µM of each dNTP, 40 mM of phosphocreatine, 4 mM ATP, 0.1 mg/ml creatine phosphokinase) for 60 min at 37°C. Beads were washed three times (150 mM NaCl, 20 mM Tris pH 7.5, 0.1% NP-40) and resuspended in Laemmli buffer before Western-Blot (WB) analysis. 

## RESULTS

### eH3.2 stably associates with chromatin and is deposited during S-phase

**Figure 1.**
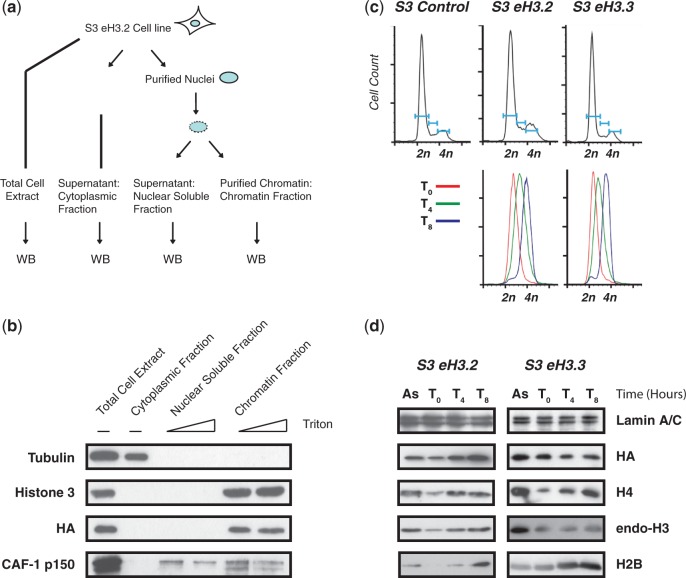
The eH3.2 incorporates into chromatin during S-phase. (**a**) Schematic diagram of the procedure used to obtain total extract, cytoplasmic fraction, nuclear soluble fraction and chromatin. (**b**) The eH3.2 stably associates with chromatin. Total extract, cytoplasmic and nuclear soluble fractions and chromatin were analysed by immunoblotting using indicated antibodies. Nuclear soluble and chromatin fractions were extracted using increasing concentrations of detergent (0.2 and 0.8% Triton X-100) as indicated in the figure. (**c**) DNA content of asynchronous control S3, S3 eH3.2 and S3 eH3.3 cells was analysed by flow cytometry (top panel). DNA content of S3 eH3.2 and S3 eH3.3 at the indicated times after release from double thymidine block (bottom panel). (**d**) Chromatin from asynchronous (As) or synchronized S3 eH3.2 and S3 eH3.3 cells harvested at 0, 4 and 8 h after release from double thymidine block was analysed by immunoblotting using the indicated antibodies.

The identification of deposition complexes for histone H3 variants 1 and 3 prompted us to investigate the complex that is involved in the deposition of the H3.2, the identity of which is currently unknown. To address this question, we established a HeLa S3 cell line, S3 eH3.2, which stably expresses histone H3 variant 2 as a C-terminal fusion protein with both Flag and HA tags (eH3.2). Next, S3 eH3.2 cells were separated into cytoplasmic, nuclear soluble and chromatin fractions (Figure 1A). The WB analysis showed that most of the tubulin remains in the cytoplasmic fraction (Figure 1B), whereas the nuclear soluble fraction is enriched in CAF-1 p150. Finally, the chromatin fraction retains most of the endogenous histone 3. Enrichment of eH3.2 was then analysed using an anti-HA antibody. As shown in Figure 1B, eH3.2 was enriched in the chromatin fraction. This association was stable, as it was not diminished at high stringency of extraction.

It is assumed that H3.2 deposition, like that of H3.1, occurs during ongoing DNA replication in contrast to the RI variant H3.3. If so, we would expect to observe an enrichment of eH3.2 associated with chromatin during S-phase of the cell cycle, when compared with eH3.3. To address this, S3 eH3.2 and S3 eH3.3 cells were synchronized by double thymidine block at the G1/S transition, then released into S-phase. DNA content was quantified by fluorescence-activated cell sorting analysis in cells that were asynchronous (As) or synchronized in early (0 h after release from G1/S), middle (4 h after release) and late S-phase (8 h after release) (Figure 1C). Incorporation of eH3.2 and eH3.3 into chromatin was monitored by WB using anti-HA antibody (Figure 1D). Association of eH3.2 with chromatin increased with DNA content, whereas eH3.3 association with chromatin was unaffected, as expected. A similar increase was observed for all histones (H2A, H2B, H4 and endogenous H3) compared with the control (Lamin A/C). These results show that eH3.2 is associated with chromatin, and that its incorporation into chromatin can occur during S-phase. Because histone incorporation into DNA is highly inefficient in the absence of chaperones or requires specific conditions in the absence of chaperones (18,19), it implies that a dedicated chaperone is involved in the deposition of eH3.2 into chromatin during S-phase.

### Identification of eH3.2 partners

**Figure 2.**
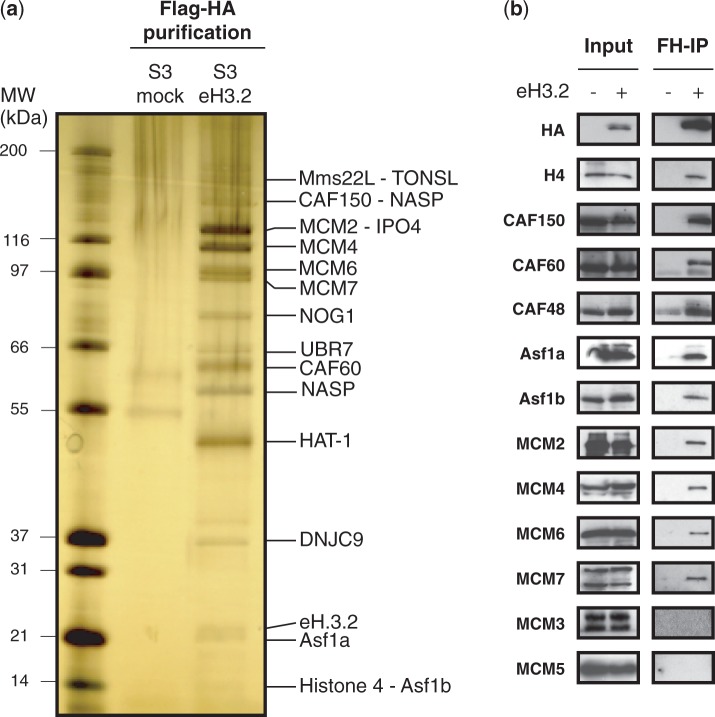
Identification of eH3.2 partners. (**a**) Silver staining of eH3.2 immunopurified partners. The NE from S3 mock or S3 eH3.2 was subjected to double Flag-HA immunopurification. A total 10% of immunopurified material was analysed by SDS–PAGE followed by silver staining. Proteins indicated on right were identified by mass spectrometry analysis**. (b**) An equal amount of NE (input) and Flag-HA-immunopurified material (FH-IP) from control S3 cells (−) or S3 cells stably expressing eH3.2 (+) were analysed by immunoblotting using the indicated antibodies.

To further characterize H3.2 deposition into chromatin, we next wished to identify the proteins involved. Previous studies have identified such complexes from NEs (17). To identify eH3.2 partners and proteins involved in its deposition, we used double affinity purification from NE followed by tandem mass spectrometry analysis*.* The NE from S3 mock or eH3.2 cells was prepared and Flag-HA purification was performed as described previously (6). Immunopurified eH3.2 (10%) was analysed by silver staining that showed the enrichment of specific proteins, which were subsequently identified by mass spectrometry (Figure 2A and Supplementary Figure S1A). Interestingly, known partners of H3.1, such as nuclear autoantigenic sperm protein (NASP), importin-4 (IPO4), tNASP, histone acetyltransferase 1 (HAT-1), Asf1a, Asf1b and H4, were identified. Two subunits, p150 and p60, of the CAF-1 complex that has been implicated in the deposition of H3.1, were also identified. Also present were MCM proteins, MCM-2, MCM-4, MCM-6 and MCM-7. We next validated these interactions by Flag-HA immunoprecipitation (IP) followed by western blot using NE from S3 mock and S3 eH3.2 (Figure 2B). CAF-1 p150 and p60 subunits identified by MS specifically interacted with eH3.2. Additionally, the p48 subunit of the trimeric CAF-1 complex that was not identified by mass spectrometry was also enriched in the eH3.2 IP. Interaction between eH3.2 and MCM-2, MCM-4, MCM-6 and MCM-7 was readily detected. The replicative helicase involved in DNA unwinding during DNA replication contains MCM2, MCM3, MCM4, MCM5 MCM6 and MCM7. MCM-3 and MCM-5 were not associated with eH3.2 either by MS identification or by WB of eH3.2 immunoprecipitate (Figure 2A andB). This result is in agreement with the previous identification of an MCM-Asf1 complex associated with H3 (20). Finally, neither histone H2A nor H2B was identified by MS. In contrast, H4 showed the highest peptide coverage (Supplementary Figure S1A), which gives a semi-quantitative analysis of an interaction. This result concurs with previous reports proposing that H3/H4 is deposited on chromatin as a tetramer (21,22).

### MCM proteins and Asf1 proteins stably interact with all histone 3 variants

**Figure 3.**
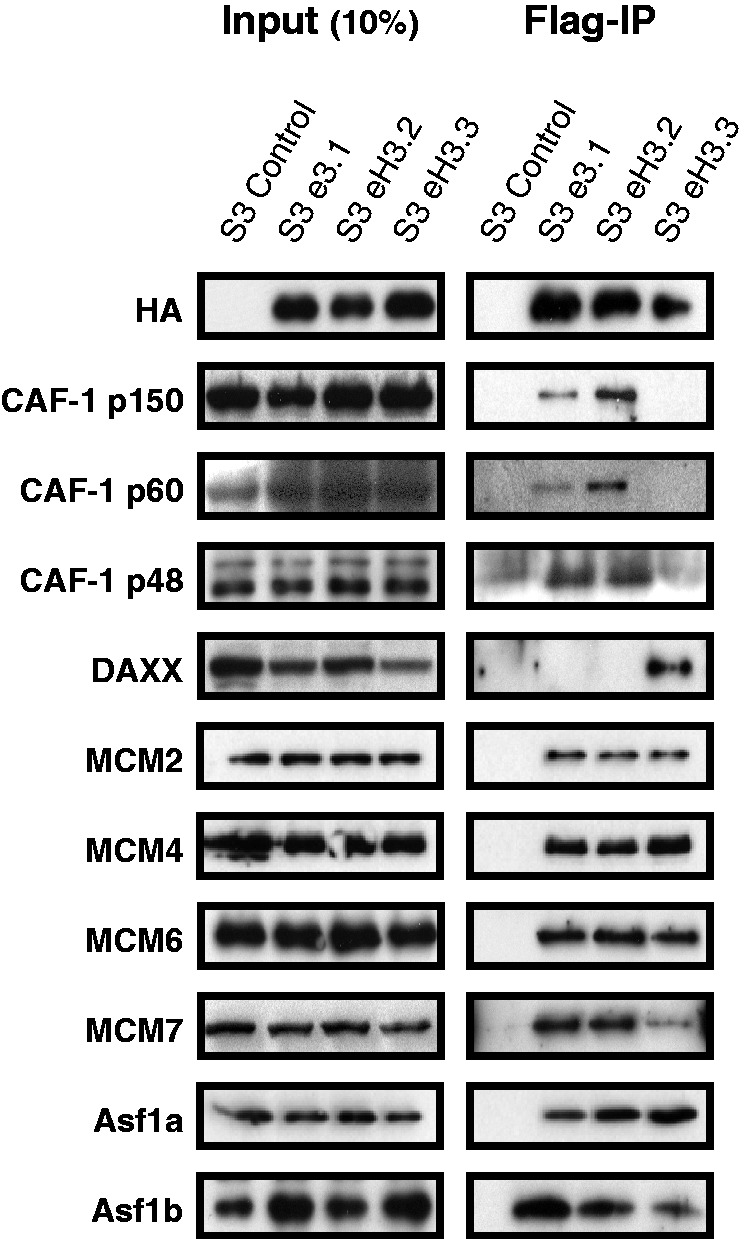
CAF-1, MCM and Asf1 proteins associate with RD histone 3 variants. Flag-IPs were performed using NE from control S3, S3-eH3.1, S3-eH3.2 and S3-eH3.3 cell lines as indicated. The NE (left panel, 10% of input material) and Flag-IPs (right panel) were resolved by SDS–PAGE and immunoblotted using the indicated antibodies.

We first analysed proteins that have previously been implicated in deposition/eviction of H3 (CAF-1, MCMs and Asf1) to address the specificity of these interactions for eH3.2. Because individual histone 3 variants cannot be distinguished by specific antibodies, we generated stable cell lines that express each histone variant as a Flag-HA epitope-tagged protein (eH3.1, eH3.2 and eH3.3; Figure 3, left panel). Flag-IPs were performed. An approximately equal amount of each histone variant was immunoprecipitated from NE (Figure 3, right panel, anti-HA). All three subunits of the CAF-1 complex (p150, p60 and p48) were enriched in eH3.1 and eH3.2 IPs but were not detected in eH3.3 IPs. In contrast, MCM 2, 4, 6 and 7 and Asf1a and b interacted with all H3 variants. As expected, DAXX interacted specifically with H3.3 (8–10).

### eH3.2 forms two major complexes within the nucleus

**Figure 4.**
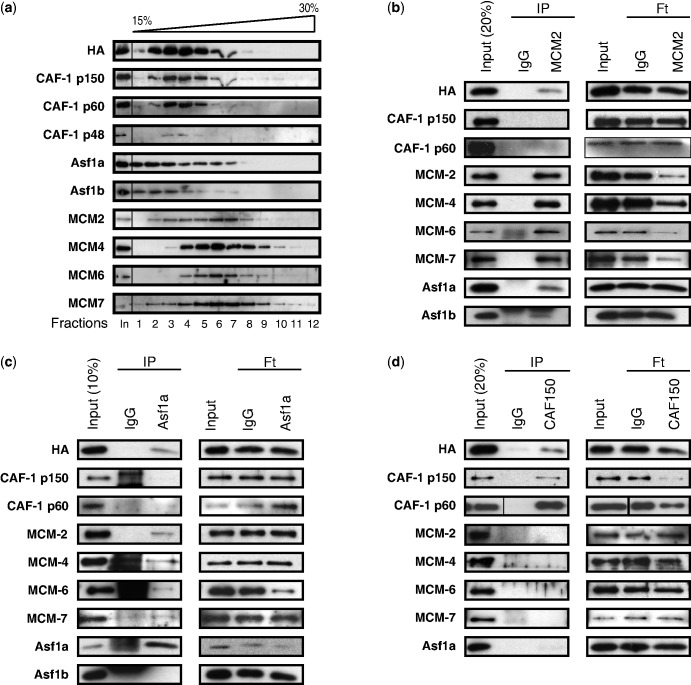
Identification of two major nuclear eH3.2 complexes. (**a**) Flag-immunopurified material from S3-eH3.2 was eluted using Flag-peptide and subjected to glycerol gradient sedimentation. An equal volume of each fraction was analysed by immunoblotting using the indicated antibodies. (**b–d**) Sequential IPs characterize two distinct complexes. Flag-immunoprecipitated material from S3-eH3.2 was subjected to a second IP using the antibodies indicated above the blot. Input, IPs and flow-through samples were analysed by immunoblotting using the indicated antibodies.

Because an interaction between CAF-1 and MCM complex has not been reported, and because eH3.3 interacted with MCM proteins but not CAF-1 (Figure 3), we hypothesized that eH3.2 may form distinct complexes within the nucleus. To address this hypothesis, eluted Flag-eH3.2 complexes were resolved by glycerol gradient sedimentation and an equal volume of each fraction was analysed by WB for eH3.2 partners (Figure 4A). The CAF-1 complex (p150, p60 and p48) co-sedimented with the major eH3.2 peak that was found in fractions 3 and 4. Both Asf1a and Asf1b were detected from fraction 1–7 with a main peak in fractions 1 and 2. Finally, MCM proteins co-sedimented with a major peak in fractions 6 and 7. This analysis suggests that eH3.2 forms a major complex with CAF-1 that may also include MCM proteins and Asf1, which readily dissociate or, alternatively, eH3.2 may form separate complexes with MCM and/or Asf1 in addition to CAF-1. Recent studies showing that H3–H4 dimers are passed from Asf1 to the CAF-1 complex via a transient interaction (21,22) would suggest eH3.2 may be present in separate complexes that contain either MCM/Asf1 or CAF1.

**Figure 5.**
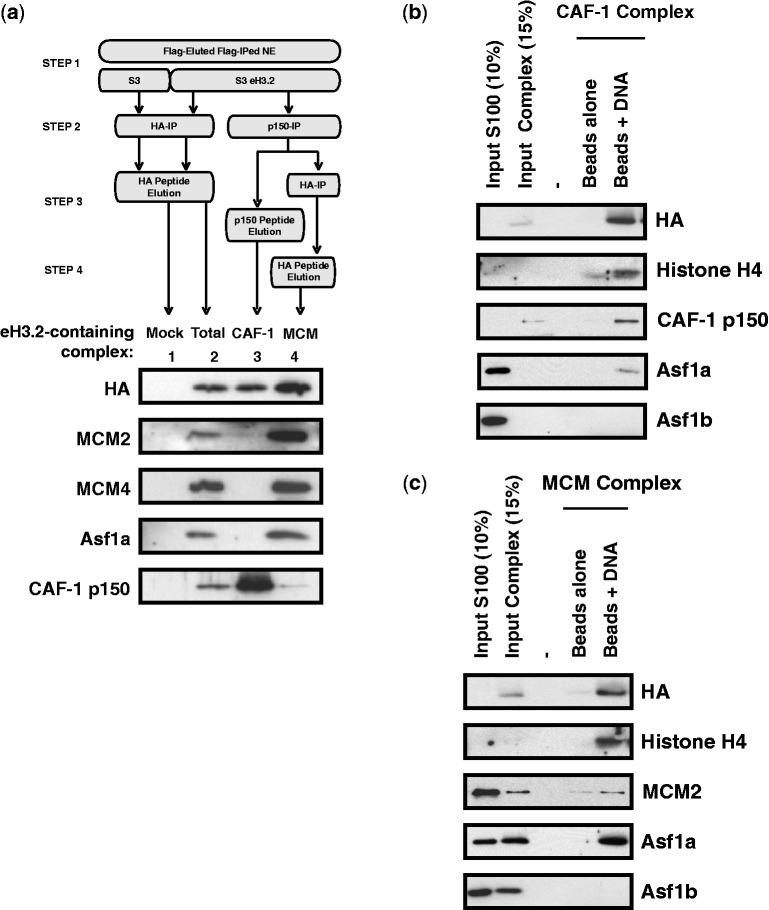
MCM/Asf1- and CAF-1-eH3.2 complexes associate with DNA *in vitro*. (**a**) Schematic representation of the experimental procedure used to isolate eH3.2 complexes containing CAF-1 or MCM/Asf1 proteins (top panel). Samples collected after each purification step were immunoblotted using the indicated antibodies (bottom panel). (**b** and **c**) *In vitro* DNA binding assays were performed with S100 extracts complemented with eluted CAF-1 complex (b) or MCM complex (c) in the presence and absence of DNA template as indicated. Inputs (S100 and purified complexes) and bound material were analysed by immunoblotting using the indicated antibodies.

To further characterize these potential complexes, we performed sequential IPs (ReIPs). Flag-purified material was subjected to a second IP using antibody to MCM2 (Figure 4B), CAF-1 p150 (Figure 4C) or Asf1a (Figure 4D). Input material (Flag-IPs), flow-through (Ft, right panel) and ReIPs (left panel, IP) were analysed by WB using the indicated antibodies. ReIP using anti-MCM2 antibody resulted in enrichment of eH3.2 (HA), MCM2, MCM4, MCM6, MCM7, Asf1a and Asf1b in the immunoprecipitates (Figure 4B). ReIP using anti-Asf1a modestly enriched eH3.2, MCM2, MCM4, MCM6 and MCM7, whereas CAF-1 p150, CAF-1 p60 and Asf1b were not significantly recovered (Figure 4C). When CAF-1 p150 ReIPs were performed, HA and CAF-1 subunits were enriched, whereas little or no signal for MCMs or Asf1 was detected (Figure 4C). Although an effect of epitope masking by the antibodies used for immunoprecipitation cannot be completely ruled out, glycerol gradient and ReIP analyses indicate that in all likelihood eH3.2 forms two complexes, one containing CAF-1 and the other containing MCM-2/4/6/7 and Asf1, although Asf1 may dissociate under certain conditions. Furthermore, Asf1a ReIP did not contain Asf1b, suggesting that the MCM/eH3.2 complex may associate with Asf1a and Asf1b separately. To further investigate eH3.2 complexes, we purified CAF-1 and MCM/Asf1 complexes separately by affinity purification strategies coupled to peptide elution (Figure 5A, top panel). Briefly, eH3.2 complexes were immunoprecipitated from NE of S3 or S3 eH3.2 cells and eluted using Flag peptide. Eluates were then subjected to a second IP using anti-CAF-1 p150 that was eluted using specific peptide. The eluate contained the CAF-1/eH3.2 complex. Unbound CAF-1-depleted material was re-immunoprecipitated using anti-HA. Elution using HA peptide yielded the MCM/Asf1/eH3.2 complex. Samples collected after each purification step were analysed by WB using the indicated antibodies (Figure 5A, lower panel). Both CAF-1 and MCMs were recovered from S3-eH3.2 following Flag-HA immunopurification (Figure 5A, lane 2) compared with control S3 cells (lane 1). For an equivalent amount of eH3.2 (anti-HA), neither MCMs nor Asf1a was detectable in CAF-1/eH3.2 immunoprecipitates (lane 3), but were recovered in the eH3.2 complex that did not associate with CAF-1 (lane 4). Taken together, eH3.2 appears to form complexes with CAF-1 and MCM-2/4/6/7/Asf1, which can be isolated separately.

We next determined whether purified CAF-1 and MCMs/Asf1 -eH3.2 complexes can associate with DNA *in vitro*. We used a previously described assay (23) that uses a biotin-labelled DNA template that was incubated using the purified complexes described in Figure 5A. The eH3.2 association with DNA was monitored by anti-HA WB (Figure 5B and C). Both CAF-1/eH3.2 (Figure 5B) and MCMs/Asf1/eH3.2 (Figure 5C) complexes associated with DNA, most likely together with histone H4. Proliferating cell nuclear antigen (PCNA), which interacts with CAF1-p150 and confers DNA synthesis-coupled specificity (24,25), was present in S100 extracts added to the reaction (data not shown). Although Asf1b was present in the reaction, no association with DNA was detected under the experimental conditions used. Thus, both CAF-1/eH3.2 and MCM/Asf1/eH3.2 complexes are competent for DNA association.

## DISCUSSION

Although the deposition complexes for non-centromeric histone variants, H3.1 and H3.3, have been identified, no biochemical data are available for the closely related variant H3.2 whose deposition is coupled to DNA synthesis, such as during DNA replication or repair. In our aim to identify the mechanism of H3.2 deposition into chromatin, we report here the first characterization of H3.2 partners from the nucleus.

Histone chaperones bind directly to histones and can direct nucleosome assembly through multiple pathways (18). We determined that eH3.2 associates with multiple histone chaperones (Figure 2) and forms two major complexes that are largely distinct (Figure 4). The most abundant is the trimeric CAF-1 complex, consisting of p150, p60 and p48 subunits (Figure 4D), whereas the minor complex contains Asf1 proteins and MCM 2/4/6/7 (Figures 4B and C). *In vitro*, eH3.2 is associated with a DNA template when bound to either CAF-1 (Figure 5B) or MCM/Asf1 (Figure 5C) complexes. Asf1 is a major H3/H4 chaperone that facilitates import of H3/H4 to the nucleus (12). It also acts as a histone ‘sink’ by buffering the majority of non-nucleosomal H3/H4 (6,26). DNA synthesis-coupled nucleosome assembly occurs via the ‘handoff’ of H3/H4 from Asf1 to the downstream CAF-1. Interaction between CAF-1 and PCNA targets H3/H4 deposition to sites of DNA synthesis, where tetrasomes are formed (6,21,27). A recent study showed that, although Asf1 and CAF-1 interact in the absence of H3-H4 dimers, specific and mutually exclusive complexes are formed in the presence of H3-H4 dimers (21). Overall, these results favour a model in which H3-H4 dimers are passed from Asf1 to the CAF-1 complex via a transient interaction leading to the formation of (H3–H4)2 tetramers within the CAF-1 complex prior its deposition onto DNA. Consistent with this view, eH3.2 complexes purified in this study were found to contain either CAF-1 or Asf1/MCM, suggesting that H3.2/H4 may also be handled in a similar manner.

The deposition of H3.1 exclusively onto newly synthesized DNA is explained by the fact that CAF-1 deposits dimers containing H3.1 but not H3.3 (7). Our data show that CAF-1 also interacts with H3.2 and associates with a DNA template together with eH3.2 *in vitro*. Consistent with previous reports, CAF-1 did not interact with H3.3. Furthermore, and consistent with previous reports (13,14), we observed that eH3.2 is deposited during S-phase (Figure 1D). Thus, the specificity of CAF-1 for H3.1 and H3.2 explains their DNA synthesis-coupled deposition.

MCM2-7 helicase has been implicated in the transfer of old histones to new DNA during replication (28). The current model of histone recycling is that old H3.1/H4 tetramers, which are retained in the vicinity of their original locus, are redeposited onto newly replicated DNA (29). Nucleosomes rarely contain a mixture of old and new H3.1/H4 (27). In support of this model, MCM2 binds to Histone 3 with high affinity (30). Interestingly, neither MCM3 nor MCM5 can bind H3, which corroborates the observation that neither MCM 3 nor MCM5 is present in the H3.2 complex. Thus, our finding that MCM/Asf1 associates with H3.2 suggests that H3.2/H4 may be recycled through a mechanism similar to that of H3.1.

In addition to the major chaperones CAF1 and Asf1, H3/H4 are first handled by upstream chaperones including HSC70, HSP90, HAT1 and NASP (31), several of which were also identified as H3.2 interacting factors (Figure 2 and Supplementary Figure S1). Interestingly, MMS22L/TONSL, which is thought to be involved in chromatin assembly at DNA-damaged forks (32–34), was also identified as a H3.2 partner (Figure 2). MMS22L was found to interact with both H3.1 and H3.2 but did not interact with H3.3 (data not shown). In summary, we have identified several complexes associated with H3.2 that may provide insight into its deposition onto DNA during DNA synthesis, for which little biochemical data are currently available.

## SUPPLEMENTARY DATA

Supplementary Data are available at NAR Online

## FUNDING

European Research Council [250333 to M.B.]; Agence Nationale de la Recherche [BLAN-0040 to M.B.]; Fondation pour la Recherche Médicale ‘équipe labellisée’ (to M.B.) and Fondation ARC pour la Recherche sur le Cancer and Fondation pour la Recherche Médicale ‘équipe labellisée’ (to R.K.); Agence Nationale de Recherches sur le Sida and Fondation pour la Recherche Médicale (to D.L.). Funding for open access charge: ERC; ANR; FRM; ANRS; and ARC.

*Conflict of interest statement*. None declared.

## Supplementary Material

Supplementary Data

## References

[1] Luger K, Mader AW, Richmond RK, Sargent DF, Richmond TJ (1997). Crystal structure of the nucleosome core particle at 2.8 A resolution. Nature.

[2] Bell O, Tiwari VK, Thoma NH, Schubeler D (2011). Determinants and dynamics of genome accessibility. Nat. Rev. Genet..

[3] Meister P, Mango SE, Gasser SM (2011). Locking the genome: nuclear organization and cell fate. Curr. Opin. Genet. Dev..

[4] Garcia BA, Hake SB, Diaz RL, Kauer M, Morris SA, Recht J, Shabanowitz J, Mishra N, Strahl BD, Allis CD (2007). Organismal differences in post-translational modifications in histones H3 and H4. J. Biol. Chem..

[5] Jung HR, Pasini D, Helin K, Jensen ON (2010). Quantitative mass spectrometry of histones H3.2 and H3.3 in Suz12-deficient mouse embryonic stem cells reveals distinct, dynamic post-translational modifications at Lys-27 and Lys-36. Mol. Cell. Proteomics.

[6] Tagami H, Ray-Gallet D, Almouzni G, Nakatani Y (2004). Histone H3.1 and H3.3 complexes mediate nucleosome assembly pathways dependent or independent of DNA synthesis. Cell.

[7] Smith S, Stillman B (1989). Purification and characterization of CAF-I, a human cell factor required for chromatin assembly during DNA replication *in vitro*. Cell.

[8] Drane P, Ouararhni K, Depaux A, Shuaib M, Hamiche A (2010). The death-associated protein DAXX is a novel histone chaperone involved in the replication-independent deposition of H3.3. Genes Dev..

[9] Goldberg AD, Banaszynski LA, Noh KM, Lewis PW, Elsaesser SJ, Stadler S, Dewell S, Law M, Guo X, Li X (2010). Distinct factors control histone variant H3.3 localization at specific genomic regions. Cell.

[10] Lewis PW, Elsaesser SJ, Noh KM, Stadler SC, Allis CD (2010). Daxx is an H3.3-specific histone chaperone and cooperates with ATRX in replication-independent chromatin assembly at telomeres. Proc. Natl Acad. Sci. USA.

[11] Ray-Gallet D, Quivy JP, Scamps C, Martini EM, Lipinski M, Almouzni G (2002). HIRA is critical for a nucleosome assembly pathway independent of DNA synthesis. Mol. Cell.

[12] Campos EI, Fillingham J, Li G, Zheng H, Voigt P, Kuo WH, Seepany H, Gao Z, Day LA, Greenblatt JF (2010). The program for processing newly synthesized histones H3.1 and H4. Nat. Struct. Mol. Biol..

[13] Akiyama T, Suzuki O, Matsuda J, Aoki F (2011). Dynamic replacement of histone H3 variants reprograms epigenetic marks in early mouse embryos. PLoS Genet..

[14] Santenard A, Ziegler-Birling C, Koch M, Tora L, Bannister AJ, Torres-Padilla ME (2010). Heterochromatin formation in the mouse embryo requires critical residues of the histone variant H3.3. Nat. Cell Biol..

[15] Nakatani Y, Ogryzko V (2003). Immunoaffinity purification of mammalian protein complexes. Methods Enzymol..

[16] Nishiyama A, Frappier L, Mechali M (2011). MCM-BP regulates unloading of the MCM2-7 helicase in late S phase. Genes Dev..

[17] Dignam JD, Lebovitz RM, Roeder RG (1983). Accurate transcription initiation by RNA polymerase II in a soluble extract from isolated mammalian nuclei. Nucleic Acids Res..

[18] Das C, Tyler JK, Churchill ME (2010). The histone shuffle: histone chaperones in an energetic dance. Trends Biochem. Sci..

[19] Germond JE, Bellard M, Oudet P, Chambon P (1976). Stability of nucleosomes in native and reconstituted chromatins. Nucleic Acids Res..

[20] Groth A, Corpet A, Cook AJ, Roche D, Bartek J, Lukas J, Almouzni G (2007). Regulation of replication fork progression through histone supply and demand. Science.

[21] Liu WH, Roemer SC, Port AM, Churchill ME (2012). CAF-1-induced oligomerization of histones H3/H4 and mutually exclusive interactions with Asf1 guide H3/H4 transitions among histone chaperones and DNA. Nucleic Acids Res..

[22] Winkler DD, Zhou H, Dar MA, Zhang Z, Luger K (2012). Yeast CAF-1 assembles histone (H3-H4)2 tetramers prior to DNA deposition. Nucleic Acids Res..

[23] Gerard A, Polo SE, Roche D, Almouzni G (2006). Methods for studying chromatin assembly coupled to DNA repair. Methods Enzymol..

[24] Gerard A, Koundrioukoff S, Ramillon V, Sergere JC, Mailand N, Quivy JP, Almouzni G (2006). The replication kinase Cdc7-Dbf4 promotes the interaction of the p150 subunit of chromatin assembly factor 1 with proliferating cell nuclear antigen. EMBO Rep..

[25] Shibahara K, Stillman B (1999). Replication-dependent marking of DNA by PCNA facilitates CAF-1-coupled inheritance of chromatin. Cell.

[26] Groth A, Ray-Gallet D, Quivy JP, Lukas J, Bartek J, Almouzni G (2005). Human Asf1 regulates the flow of S phase histones during replicational stress. Mol. Cell.

[27] Xu M, Long C, Chen X, Huang C, Chen S, Zhu B (2010). Partitioning of histone H3-H4 tetramers during DNA replication-dependent chromatin assembly. Science.

[28] Groth A (2009). Replicating chromatin: a tale of histones. Biochem. Cell Biol..

[29] Alabert C, Groth A (2012). Chromatin replication and epigenome maintenance. Nat. Rev. Mol. Cell Biol..

[30] Ishimi Y, Ichinose S, Omori A, Sato K, Kimura H (1996). Binding of human minichromosome maintenance proteins with histone H3. J. Biol. Chem..

[31] Campos EI, Fillingham J, Li G, Zheng H, Voigt P, Kuo WH, Seepany H, Gao Z, Day LA, Greenblatt JF (2010). The program for processing newly synthesized histones H3.1 and H4. Nat. Struct. Mol. Biol..

[32] O'Connell BC, Adamson B, Lydeard JR, Sowa ME, Ciccia A, Bredemeyer AL, Schlabach M, Gygi SP, Elledge SJ, Harper JW (2010). A genome-wide camptothecin sensitivity screen identifies a mammalian MMS22L-NFKBIL2 complex required for genomic stability. Mol. Cell.

[33] O'Donnell L, Panier S, Wildenhain J, Tkach JM, Al-Hakim A, Landry MC, Escribano-Diaz C, Szilard RK, Young JT, Munro M (2010). The MMS22L-TONSL complex mediates recovery from replication stress and homologous recombination. Mol. Cell.

[34] Piwko W, Olma MH, Held M, Bianco JN, Pedrioli PG, Hofmann K, Pasero P, Gerlich DW, Peter M (2010). RNAi-based screening identifies the Mms22L-Nfkbil2 complex as a novel regulator of DNA replication in human cells. EMBO J..

