# Supplementary material for: Identification of histone 3 variant 2 interacting factors
Source: Nucleic Acids Res. 2014 Jan 6;42(6):3542–50. doi: 10.1093/nar/gkt1355 (PMC3973350; doi:10.1093/nar/gkt1355)
Supplement: Supplementary Data [file supp_42_6_3542__index.html]

Identification of histone 3 variant 2 interacting factors — Identification of histone 3 variant 2 interacting factors — Supplementary Data 

# Identification of histone 3 variant 2 interacting factors

## Supplementary Data

files

**Files in this Data Supplement:**

- Supplementary Data - pdf file
