# Supplementary material for: Identification of histone 3 variant 2 interacting factors
Source: Nucleic Acids Res. 2014 Jan 6;42(6):3542–50. doi: 10.1093/nar/gkt1355 (PMC3973350; doi:10.1093/nar/gkt1355)
Supplement: Supplementary Data [file supp_gkt1355_nar-02103-m-2013-File003.pdf]

Peptide Coverage

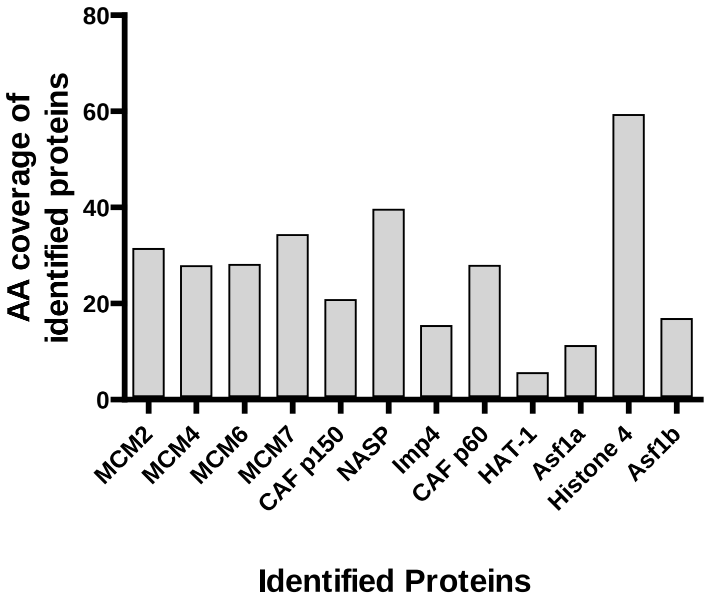

Supplementary Figure Legend

Supplementary Figure 1. Peptide coverage of eH3.2 partners identified by mass spectrometry. Identification of eH3.2 interacting factors. Shown is percent coverage of peptides identified by mass spectrometry.
